# Association of novel triglyceride-glucose index-derived indices with hyperuricemia among oilfield workers

**DOI:** 10.3389/fnut.2026.1766524

**Published:** 2026-02-06

**Authors:** Haobiao Liu, Qingsong Li, Husna Wali, Yingjie Cai, Jing Tang, Licheng Yang, Xuefeng Yu, Jinsong Li, Bowei Yang, Ziwei Guo, Jing Han, Zhiyong Du

**Affiliations:** 1 Xi'an Gem Flower Changqing Hospital, Xi'an, Shaanxi, China; 2 School of Public Health, Health Science Center, Xi'an Jiaotong University, Xi'an, Shaanxi, China

**Keywords:** hyperuricemia, insulin resistance, occupational health, oilfield worker, triglyceride-glucose index

## Abstract

**Objectives:**

The association and predictive value of newer triglyceride-glucose (TyG)-derived anthropometric indices for hyperuricemia have not been systematically evaluated.

**Methods:**

Logistic regression, restricted cubic spline (RCS) modeling, subgroup analyses, and multiple sensitivity assessments were used to evaluate dose-response patterns and the robustness of findings. Discriminatory performance was further assessed using receiver operating characteristic curves.

**Results:**

All five TyG-derived indicators showed positive associations with hyperuricemia. Each one-standard deviation increase in triglyceride-glucose-a body shape index (TyG-ABSI), triglyceride-glucose-weight-adjusted waist index (TyG-WWI), triglyceride-glucose-conicity index (TyG-CI), triglyceride-glucose-body roundness index (TyG-BRI), and triglyceride-glucose-Chinese visceral adiposity index (TyG-CVAI) corresponded to 62, 45, 68, 82, and 88% higher odds, respectively. Similar trends appeared in categorical analyses, where the highest quartile exhibited markedly elevated risks compared with the lowest, ranging from 1.61-fold for TyG-WWI to 2.29-fold for TyG-CVAI. RCS analyses confirmed non-linear patterns, and subgroup findings across age, sex, and shift-work strata were largely consistent. Sensitivity analyses further supported the robustness of the results. Among all indicators, TyG-CVAI provided the greatest discriminative ability, with an AUC of 0.735 and statistically superior performance relative to the other indices.

**Conclusion:**

TyG-derived anthropometric metrics, particularly TyG-CVAI, exhibit robust associations with hyperuricemia and demonstrate promising utility for metabolic risk stratification.

## 1 Introduction

Hyperuricemia, an elevation of serum uric acid, has grown to be a common metabolic condition with a rising incidence worldwide (1). According to the U. S. National Health and Nutrition Examination Survey data, hyperuricemia affects 21.4% of adults in the U. S. (2). In China, the prevalence increased from 8.4% in 2009–2010 to more than 11% in 2015–2016. By 2018–2019, hyperuricemia was estimated to affect approximately 14% of adults, corresponding to more than 180 million individuals nationwide (3, 4). Due to this increase, hyperuricemia has been dubbed the “fourth high” in China’s public health landscape, along with hypertension, hyperlipidemia, and hyperglycemia. Certain high-risk occupational groups exhibit even greater prevalence; for example, a recent large-scale survey of Chinese oilfield workers reported that 24.5% had hyperuricemia (5), exposing a developing health problem in this population. These patterns are concerning because hyperuricemia is a precursor to gout and urate kidney stones, and it is also closely linked to cardiovascular, renal, and other metabolic comorbidities (6). With hyperuricemia becoming more common and affecting younger people more frequently (3), to reduce long-term organ damage and socioeconomic burden, early detection of at-risk individuals is crucial.

Insulin resistance and obesity are known risk factors for hyperuricemia (7, 8). Mechanistically, renal uric acid excretion can be decreased by insulin resistance and compensatory hyperinsulinemia, raising serum uric acid levels (9). On the other hand, increased uric acid may worsen endothelial dysfunction and oxidative stress, indicating a reciprocal relationship within metabolic syndrome (10). Because of this intimate interaction, insulin resistance has been identified as a major factor in the pathophysiology of hyperuricemia (11). In this regard, the triglyceride-glucose (TyG) index, which is derived from fasting glucose and triglyceride levels, has become a straightforward and trustworthy proxy for insulin resistance (12). Higher TyG index values are positively correlated with uric acid levels and the risk of hyperuricemia, according to an increasing amount of research. People in the highest TyG index quartile, for example, had more than twice the odds of hyperuricemia compared to those in the lowest quartile, according to a recent national analysis (13). In a similar vein, other research has discovered that in a variety of populations, the TyG index is more closely linked to hyperuricemia than conventional obesity measures (14). These results show that TyG is a promising metabolic marker for identifying people who are at risk for hyperuricemia.

However, adiposity and fat distribution, two crucial aspects of metabolic health and urate metabolism, are not captured by the TyG index alone (15). Obesity is linked to an increased risk of hyperuricemia because excess adiposity, especially visceral fat, is known to increase uric acid production and impair its excretion (14). Conventional anthropometric indices, like body mass index (BMI) and waist circumference (WC), are unable to differentiate between visceral and subcutaneous fat depots and only offer imprecise indicators of obesity. In fact, BMI and WC frequently overlook subtleties in the distribution of body fat (16, 17). In order to overcome these constraints, a number of new anthropometric indices that more accurately reflect body composition and fat distribution have been developed. Indicators such as a body shape index (ABSI), weight-adjusted waist index (WWI), conicity index (CI), body roundness index (BRI), and Chinese visceral adiposity index (CVAI) integrate information from weight, height, BMI, and waist circumference to better capture visceral adiposity and related metabolic abnormalities. Compared with traditional measures such as BMI or WC, these newer indices demonstrate stronger associations with various components of metabolic syndrome (18).

Almost no research has examined the associations of the recently proposed TyG-derived composite indices: TyG-ABSI, TyG-WWI, TyG-CI, TyG-BRI, and TyG-CVAI in relation to the risk of hyperuricemia. This disparity is especially noticeable in occupational cohorts like oilfield workers, who may have particular lifestyle factors that affect metabolic health, such as intense labor, dietary habits, and shift schedules. Understanding whether these novel TyG-derived indicators can identify people at elevated risk is crucial for both clinical and preventive purposes, given the high and increasing prevalence of hyperuricemia seen in oilfield workers (5).

Thus, the purpose of this study was to investigate the association between hyperuricemia in oilfield workers and a set of novel TyG-derived composite indices (TyG-ABSI, TyG-WWI, TyG-CI, TyG-BRI, and TyG-CVAI). The study intends to fill a research gap and offer a theoretical foundation for incorporating metabolic and anthropometric markers into hyperuricemia risk assessment by determining which of these indices most strongly correlates with hyperuricemia. In the end, the results might help identify high-risk people in oilfield environments earlier and direct more focused preventative measures to lessen the growing prevalence of hyperuricemia.

## 2 Materials and methods

### 2.1 Study design and population

**Figure 1:**
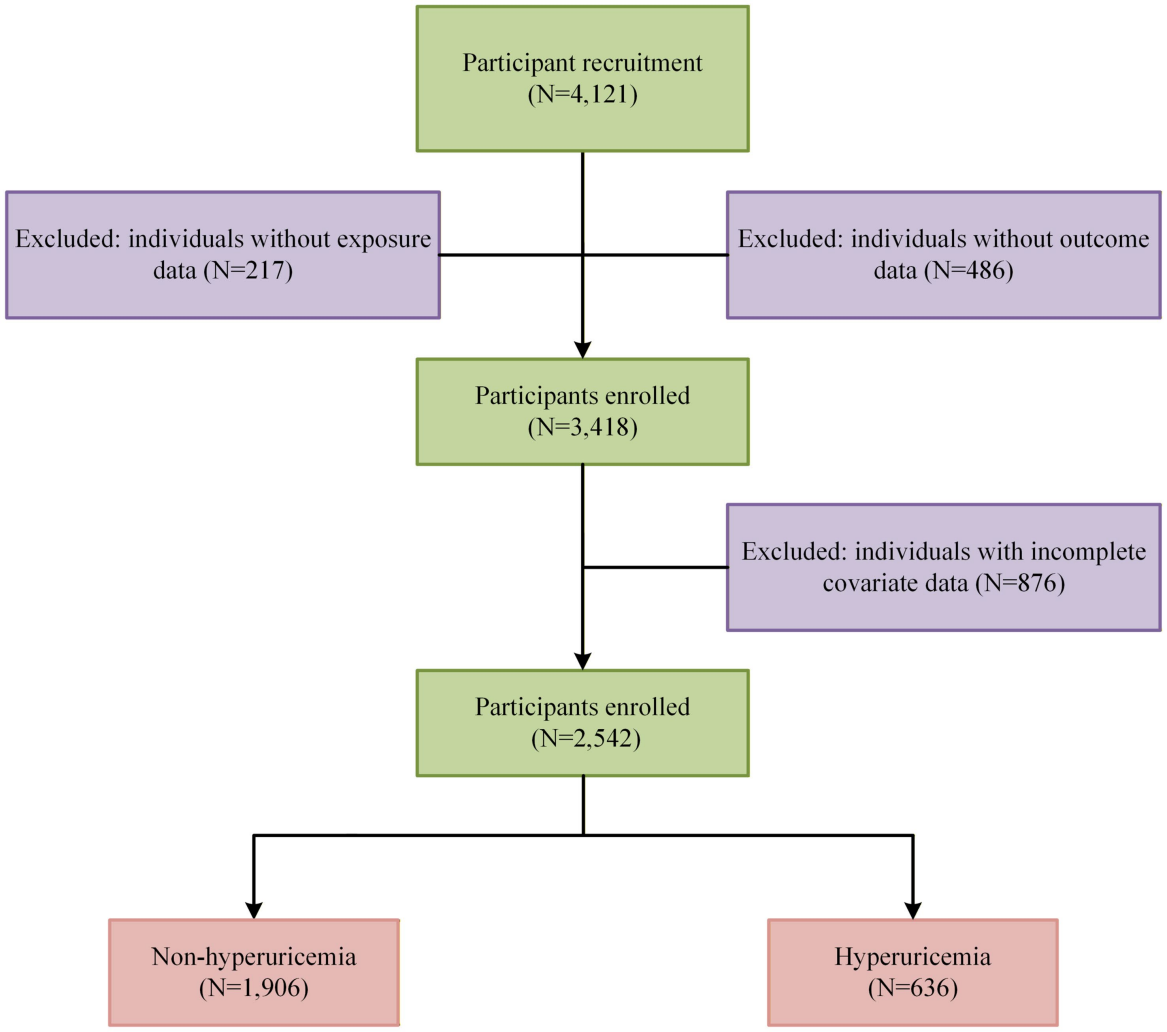
Flow diagram of participants included in this study.

This study was conducted among employees of a petroleum enterprise in Xi’an, Shaanxi Province, China. Participant recruitment took place at the enterprise-affiliated hospital between October and December 2022. The study population included frontline oilfield workers (e.g., drilling, extraction, and maintenance staff), as well as technical, administrative, and logistics personnel within the enterprise. During on-site health examination visits, trained investigators administered face-to-face questionnaires, and anthropometric and laboratory data were obtained from the hospital’s electronic medical record system. Eligible participants were required to be aged 18 years or older, have been employed for at least 1 year, and voluntarily agree to participate. Individuals were excluded if they were pregnant or breastfeeding, had severe neurological or psychiatric disorders that could impede completion of the survey. A total of 4,121 employees were initially screened. Of these, 217 were excluded due to missing exposure variables, 486 due to missing outcome data, and 876 due to incomplete covariate information. After applying all inclusion and exclusion criteria, 2,542 participants were included in the final analyses (Figure 1). All participants provided written informed consent before enrollment. The study protocol was reviewed and approved by the Medical Ethics Committee of Xi’an Jiaotong University, and all procedures adhered to the ethical principles of the Declaration of Helsinki.

### 2.2 Assessment of TyG-derived indices

All anthropometric measurements and fasting blood samples, including triglycerides, fasting plasma glucose, and serum uric acid, were obtained on the same day during standardized health examinations. Fasting venous blood samples were obtained after at least 8 h of overnight fasting. Serum triglyceride and fasting plasma glucose concentrations were measured using standardized enzymatic methods on an automated chemistry analyzer (Cobas 6000, Roche Diagnostics, Mannheim, Germany). Five TyG–derived indices were computed to characterize various aspects of adiposity and body composition. These included the TyG-ABSI, TyG-WWI, TyG-CI, TyG-BRI, and TyG-CVAI. All indicators were derived from anthropometric measures, including height, weight, waist circumference, and body mass index. To allow for a comprehensive evaluation of predictive capability, four commonly used TyG-related metrics were also calculated: the TyG, TyG-BMI, TyG-WC, and TyG-WHtR. Detailed computational formulas for all TyG-derived indices are presented in Supplementary Table S1.

### 2.3 Definition of hyperuricemia

Venous blood samples were obtained from the antecubital vein in the morning after an overnight fast. Serum uric acid concentrations were measured using the uricase–peroxidase enzymatic colorimetric method on an automated biochemical analyzer (Cobas 6000, Roche Diagnostics, Mannheim, Germany). Hyperuricemia was defined using sex-specific thresholds, with serum uric acid levels greater than 420 μmol/L (7.0 mg/dL) in male participants and greater than 360 μmol/L (6.0 mg/dL) in female participants (19).

### 2.4 Covariates

Potential confounding variables were selected based on prior epidemiological evidence and biological plausibility (20). These included demographic characteristics (age, sex, ethnicity, marital status, education level, annual income), occupational factors (shift work, exposure to chemical substances, noise, and dust), lifestyle behaviors (smoking status, alcohol drinking, physical activity, and tea consumption), clinical conditions (hypertension, dyslipidemia, and cardiovascular disease), and kidney function assessed by estimated glomerular filtration rate (eGFR). Detailed definitions and measurement procedures of all covariates are presented in Supplementary Table S2.

### 2.5 Statistical analysis

All statistical analyses were conducted using R software (version 4.4.0). Continuous variables were presented as means ± standard deviations (SD), whereas categorical variables were summarized as counts and percentages. Differences between participants with and without hyperuricemia were evaluated using the Student’s *t*-test for continuous variables and the chi-square test for categorical variables.

Binary logistic regression models were applied to estimate the associations between TyG-derived indices and hyperuricemia, with results presented as odds ratios (ORs) and 95% confidence intervals (CIs). Each TyG-derived indicator was examined both as a standardized continuous variable (per SD increase) and as a categorical variable based on quartiles. Linear trend tests were performed by assigning the median value of each quartile and modeling it as a continuous variable. Three hierarchical models were developed to progressively control for potential confounders. Model 1 included no covariates. Model 2 adjusted for demographic characteristics. Model 3 further accounted for occupational exposures, lifestyle behaviors, clinical factors, and kidney function. Restricted cubic spline (RCS) analyses were performed using three knots placed at the 10th, 50th, and 90th percentiles of each TyG-derived index to evaluate potential non-linear dose–response relationships.

Subgroup analyses were stratified by age, sex, and shift-work status to assess potential effect modification. Receiver operating characteristic (ROC) curve analyses were used to assess the ability of each TyG-derived index (TyG-ABSI, TyG-WWI, TyG-CI, TyG-BRI, and TyG-CVAI) to discriminate hyperuricemia. For comparison, four conventional TyG-related indicators (TyG-BMI, TyG-WC, TyG-WHtR, and TyG) were also evaluated. The area under the curve (AUC) was calculated for each indicator, and pairwise comparisons were conducted using the DeLong method. Additionally, net reclassification improvement (NRI) and integrated discrimination improvement (IDI) analyses were performed to further quantify the improvement in predictive performance between the optimal TyG-derived index and the TyG index. Multiple sensitivity analyses were performed to verify the robustness of the findings, including (1) multiple imputation for missing covariates; (2) applying the Chinese clinical practice guideline definition of hyperuricemia as serum uric acid greater than 420 μmol/L; and (3) exclusion of participants with extreme values exceeding three SDs from the corresponding mean. All statistical tests were two-sided, and a *p* value <0.05 was considered statistically significant.

## 3 Results

### 3.1 Baseline characteristics of participants

A total of 2,542 participants were included in the final analysis, comprising 1,906 individuals without hyperuricemia and 636 with hyperuricemia. The baseline characteristics of the study population are summarized in Table 1. Significant differences were observed between the two groups across multiple sociodemographic, lifestyle, and clinical variables.

**Table 1:** Baseline characteristics of participants.

Variables	Total (*N* = 2,542)	Non-hyperuricemia (*N* = 1,906)	Hyperuricemia (*N* = 636)	*P* value
Age, year	41.27 (8.29)	41.97 (7.95)	39.17 (8.91)	<0.001
Sex				<0.001
Male	1,657 (65.18)	1,107 (58.08)	550 (86.48)	
Female	885 (34.82)	799 (41.92)	86 (13.52)	
Ethnicity				0.936
Han	2,493 (98.07)	1,870 (98.11)	623 (97.96)	
Other	49 (1.93)	36 (1.89)	13 (2.04)	
Education level				<0.001
High school or below	945 (37.18)	759 (39.82)	186 (29.25)	
College degree	926 (36.43)	687 (36.04)	239 (37.58)	
University graduate or above	671 (26.40)	460 (24.13)	211 (33.18)	
Marital status				<0.001
Married	2,147 (84.46)	1,661 (87.15)	486 (76.42)	
Unmarried/Separated	395 (15.54)	245 (12.85)	150 (23.58)	
Annual income, thousand (CNY)				<0.001
≤100	484 (19.04)	396 (20.78)	88 (13.84)	
101–150	1,556 (61.21)	1,160 (60.86)	396 (62.26)	
≥151	502 (19.75)	350 (18.36)	152 (23.90)	
Shift work				0.006
No	751 (29.54)	535 (28.07)	216 (33.96)	
Yes	1,791 (70.46)	1,371 (71.93)	420 (66.04)	
Chemical substance exposure				0.525
No	531 (20.89)	392 (20.57)	139 (21.86)	
Yes	2,011 (79.11)	1,514 (79.43)	497 (78.14)	
Noise exposure				0.754
No	956 (37.61)	713 (37.41)	243 (38.21)	
Yes	1,586 (62.39)	1,193 (62.59)	393 (61.79)	
Dust exposure				0.682
No	1,908 (75.06)	1,435 (75.29)	473 (74.37)	
Yes	634 (24.94)	471 (24.71)	163 (25.63)	
Cigarette smoking				<0.001
No	1,373 (54.01)	1,116 (58.55)	257 (40.41)	
Yes	1,169 (45.99)	790 (41.45)	379 (59.59)	
Alcohol drinking				<0.001
No	1,591 (62.59)	1,277 (67.00)	314 (49.37)	
Yes	951 (37.41)	629 (33.00)	322 (50.63)	
Tea drinking				0.014
No	1,216 (47.84)	939 (49.27)	277 (43.55)	
Yes	1,326 (52.16)	967 (50.73)	359 (56.45)	
Physical activity				0.291
Inactive	613 (24.11)	470 (24.66)	143 (22.48)	
Active	1,929 (75.89)	1,436 (75.34)	493 (77.52)	
Hypertension				<0.001
No	1,949 (76.67)	1,517 (79.59)	432 (67.92)	
Yes	593 (23.33)	389 (20.41)	204 (32.08)	
Hyperlipidemia				<0.001
No	1,475 (58.03)	1,230 (64.53)	245 (38.52)	
Yes	1,067 (41.97)	676 (35.47)	391 (61.48)	
Cardiovascular disease				0.971
No	2,349 (92.41)	1,762 (92.44)	587 (92.30)	
Yes	193 (7.59)	144 (7.56)	49 (7.70)	
eGFR, mL/min/1.73 m^2^	109.87 (10.84)	110.37 (10.11)	108.35 (12.69)	<0.001

Participants with hyperuricemia were generally younger and predominantly male. Compared with the non-hyperuricemia group, individuals with hyperuricemia were more likely to have attained higher educational levels and report higher annual income (*p* < 0.001). The prevalence of smoking, alcohol consumption, and tea drinking was also substantially higher among participants with hyperuricemia (*p* < 0.05). The hyperuricemia group also exhibited a greater burden of hypertension and hyperlipidemia, and a lower level of eGFR (*p* < 0.001). In contrast, no significant differences were found in ethnicity, chemical exposure, noise exposure, dust exposure, physical activity, or cardiovascular disease.

### 3.2 Associations between TyG-derived indices and hyperuricemia

Table 2 presents the associations of TyG-derived anthropometric indices with hyperuricemia risk. Across all five indices, positive associations with hyperuricemia were consistently observed in Model 1, and the effect estimates remained stable after adjustment for demographic factors in Model 2. After further adjustment for all potential confounders in Model 3, these associations persisted and were statistically significant.

**Table 2:** Associations between TyG-derived indices and the risk of hyperuricemia among oilfield workers.

Variable	Model 1		Model 2		Model 3	
	OR (95% CI)	*P* value	OR (95% CI)	*P* value	OR (95% CI)	*P* value
TyG-ABSI						
Per SD increase	1.84 (1.68, 2.03)	<0.001	1.73 (1.56, 1.92)	<0.001	1.62 (1.43, 1.85)	<0.001
Quantile 1	1.00 (Reference)		1.00 (Reference)		1.00 (Reference)	
Quantile 2	1.84 (1.33, 2.57)	<0.001	1.67 (1.18, 2.38)	0.004	1.64 (1.16, 2.35)	0.006
Quantile 3	3.86 (2.86, 5.29)	<0.001	3.65 (2.62, 5.12)	<0.001	3.39 (2.39, 4.84)	<0.001
Quantile 4	6.36 (4.73, 8.65)	<0.001	5.44 (3.92, 7.64)	<0.001	4.76 (3.21, 7.13)	<0.001
*P* for trend		<0.001		<0.001		<0.001
TyG-WWI						
Per SD increase	1.70 (1.55, 1.87)	<0.001	1.60 (1.45, 1.78)	<0.001	1.45 (1.28, 1.65)	<0.001
Quantile 1	1.00 (Reference)		1.00 (Reference)		1.00 (Reference)	
Quantile 2	1.83 (1.33, 2.51)	<0.001	1.68 (1.21, 2.36)	0.002	1.57 (1.12, 2.22)	0.009
Quantile 3	3.52 (2.63, 4.77)	<0.001	3.23 (2.35, 4.48)	<0.001	2.87 (2.05, 4.04)	<0.001
Quantile 4	4.97 (3.73, 6.70)	<0.001	4.31 (3.14, 5.97)	<0.001	3.29 (2.26, 4.83)	<0.001
*P* for trend		<0.001		<0.001		<0.001
TyG-CI						
Per SD increase	1.89 (1.72, 2.08)	<0.001	1.77 (1.60, 1.97)	<0.001	1.68 (1.47, 1.91)	<0.001
Quantile 1	1.00 (Reference)		1.00 (Reference)		1.00 (Reference)	
Quantile 2	1.91 (1.37, 2.69)	<0.001	1.77 (1.24, 2.53)	0.002	1.74 (1.22, 2.50)	0.003
Quantile 3	4.18 (3.07, 5.75)	<0.001	3.91 (2.80, 5.54)	<0.001	3.72 (2.62, 5.36)	<0.001
Quantile 4	7.09 (5.25, 9.71)	<0.001	6.13 (4.39, 8.66)	<0.001	5.55 (3.72, 8.37)	<0.001
*P* for trend		<0.001		<0.001		<0.001
TyG-BRI						
Per SD increase	2.01 (1.82, 2.22)	<0.001	1.89 (1.70, 2.11)	<0.001	1.82 (1.60, 2.08)	<0.001
Quantile 1	1.00 (Reference)		1.00 (Reference)		1.00 (Reference)	
Quantile 2	2.11 (1.49, 3.01)	<0.001	2.22 (1.54, 3.24)	<0.001	2.16 (1.48, 3.18)	<0.001
Quantile 3	4.49 (3.25, 6.29)	<0.001	4.78 (3.35, 6.91)	<0.001	4.73 (3.26, 6.97)	<0.001
Quantile 4	9.64 (7.04, 13.41)	<0.001	9.40 (6.63, 13.54)	<0.001	9.22 (6.15, 14.00)	<0.001
*P* for trend		<0.001		<0.001		<0.001
TyG-CVAI						
Per SD increase	2.11 (1.91, 2.33)	<0.001	1.95 (1.74, 2.18)	<0.001	1.88 (1.64, 2.15)	<0.001
Quantile 1	1.00 (Reference)		1.00 (Reference)		1.00 (Reference)	
Quantile 2	2.02 (1.42, 2.91)	<0.001	2.12 (1.45, 3.13)	<0.001	1.94 (1.32, 2.89)	<0.001
Quantile 3	4.72 (3.42, 6.63)	<0.001	4.89 (3.38, 7.18)	<0.001	4.34 (2.93, 6.50)	<0.001
Quantile 4	9.96 (7.27, 13.90)	<0.001	9.55 (6.58, 14.09)	<0.001	8.49 (5.52, 13.23)	<0.001
*P* for trend		<0.001		<0.001		<0.001

Model 1, no covariate was adjusted. Model 2, adjusted for age, sex, ethnicity, education level, marital status, and annual income. Model 3, further adjusted for shift work, chemical substance exposure, noise exposure, dust exposure, cigarette smoking, alcohol drinking, tea drinking, physical activity, estimated glomerular filtration rate, hypertension, hyperlipidemia, and cardiovascular disease. OR, odds ratio; CI, confidence interval.

When treated as continuous variables, each one–SD increase in the TyG-derived indices corresponded to substantial elevations in hyperuricemia risk. Specifically, a one–SD rise in TyG-ABSI was associated with a 62% higher risk of hyperuricemia (OR = 1.62, 95% CI: 1.43–1.85, *p* < 0.001), while TyG-WWI and TyG-CI were linked to 45% (OR = 1.45, 95% CI: 1.28–1.65, *p* < 0.001) and 68% (OR = 1.68, 95% CI: 1.47–1.91, *p* < 0.001) higher risks, respectively. Even steeper increments were observed for TyG-BRI and TyG-CVAI, which were associated with 82% (OR = 1.82, 95% CI: 1.60–2.08, *p* < 0.001) and 88% (OR = 1.88, 95% CI: 1.64–2.15, *p* < 0.001) higher risks, respectively.

Analyses based on quartiles further demonstrated a graded increase in risk. Compared with participants in the lowest quartile, those in the second, third, and fourth quartiles exhibited progressively higher odds of hyperuricemia. For TyG-ABSI, the adjusted ORs were 1.64 (95% CI: 1.16–2.35, *p* = 0.006), 3.39 (95% CI: 2.39–4.84, *p* < 0.001), and 4.76 (95% CI: 3.21–7.13, *p* < 0.001), respectively. Corresponding ORs for TyG-WWI were 1.57 (95% CI: 1.12–2.22, *p* = 0.009), 2.87 (95% CI: 2.05–4.04, *p* < 0.001), and 3.29 (95% CI: 2.26–4.83, *p* < 0.001). For TyG-CI, the second through fourth quartiles showed adjusted ORs of 1.74 (95% CI: 1.22–2.50, *p* = 0.003), 3.72 (95% CI: 2.62–5.36, *p* < 0.001), and 5.55 (95% CI: 3.72–8.37, *p* < 0.001). TyG-BRI displayed even stronger gradients, with ORs of 2.16 (95% CI: 1.48–3.18, *p* < 0.001), 4.73 (95% CI: 3.26–6.97, *p* < 0.001), and 9.22 (95% CI: 6.15–14.00, *p* < 0.001). For TyG-CVAI, the adjusted ORs were 1.94 (95% CI: 1.32–2.89, *p* < 0.001), 4.34 (95% CI: 2.93–6.50, *p* < 0.001), and 8.49 (95% CI: 5.52–13.23, *p* < 0.001) across ascending quartiles. For all indices, the probability of hyperuricemia increased monotonically across quartiles, and statistical tests confirmed significant linear trends across categories (all *P* for trend < 0.001).

**Figure 2:**
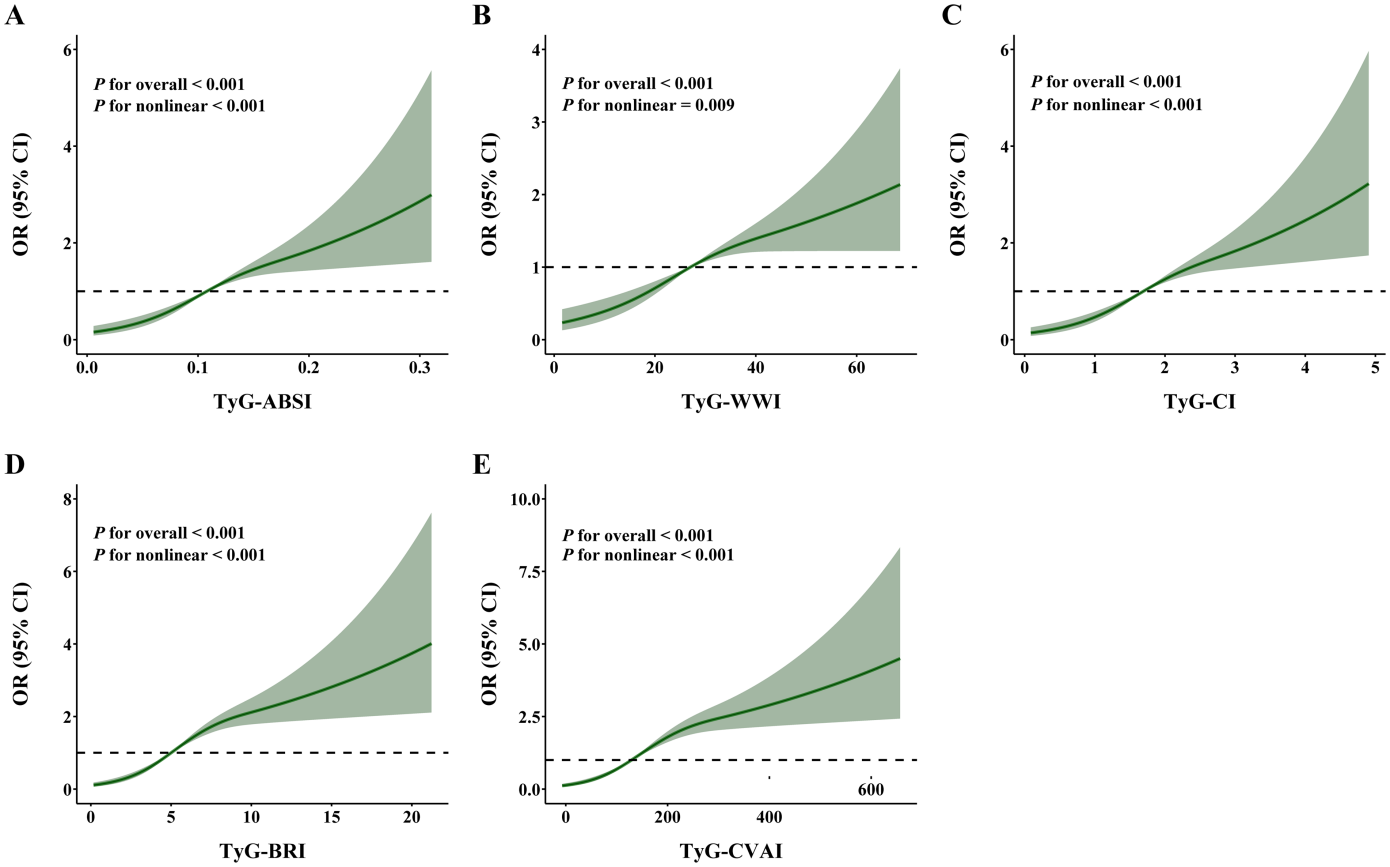
Restricted cubic spline analyses of the associations between TyG-derived indices and the risk of hyperuricemia among oilfield workers **(A–E)**. **(A)** TyG-ABSI. **(B)** TyG-WWI. **(C)** TyG-CI. **(D)** TyG-BRI. **(E)** TyG-CVAI. The models were adjusted for age, sex, ethnicity, education level, marital status, annual income, chemical substance exposure, noise exposure, dust exposure, cigarette smoking, alcohol drinking, tea drinking, physical activity, estimated glomerular filtration rate, hypertension, hyperlipidemia, and cardiovascular disease.

RCS analyses further characterized the exposure–response relationships for the five TyG-derived indices (Figure 2). All spline models revealed statistically significant non-linear associations with hyperuricemia (all *P* for nonlinear < 0.01). Overall, the predicted probability of hyperuricemia increased progressively with higher index values. These consistent non-linear patterns provide additional evidence for complex dose–response relationships for all TyG-derived indices.

### 3.3 Subgroup analyses

Stratified analyses by age, sex, and shift work status consistently demonstrated positive associations between all TyG-derived indices and hyperuricemia. Among participants younger than 40 years, the associations were generally strong, with ORs ranging from 1.34 to 2.18, whereas similar magnitudes were observed in those aged 40 years or older, with ORs spanning 1.48 to 1.64 (Supplementary Table S3). These findings suggest that age did not materially alter the relationships between TyG-related adiposity indicators and hyperuricemia risk. Comparable patterns were observed when stratified by sex. All five indices were significantly associated with hyperuricemia in both males and females (Supplementary Table S4). The overall direction and significance of associations were highly consistent across sexes. Analyses by shift work status showed that both shift and non-shift workers exhibited robust positive associations, with somewhat larger effect estimates observed among non-shift workers compared with shift workers (Supplementary Table S5). Despite minor variations in magnitude, the associations remained statistically significant in all groups. Overall, the subgroup analyses revealed no evidence of substantial effect modification by age, sex, or shift work. The consistent pattern across all strata indicates that the relationships between TyG-derived indices and hyperuricemia are broadly stable across demographic and occupational subpopulations.

### 3.4 Predictive performance of TyG-derived indices

**Figure 3:**
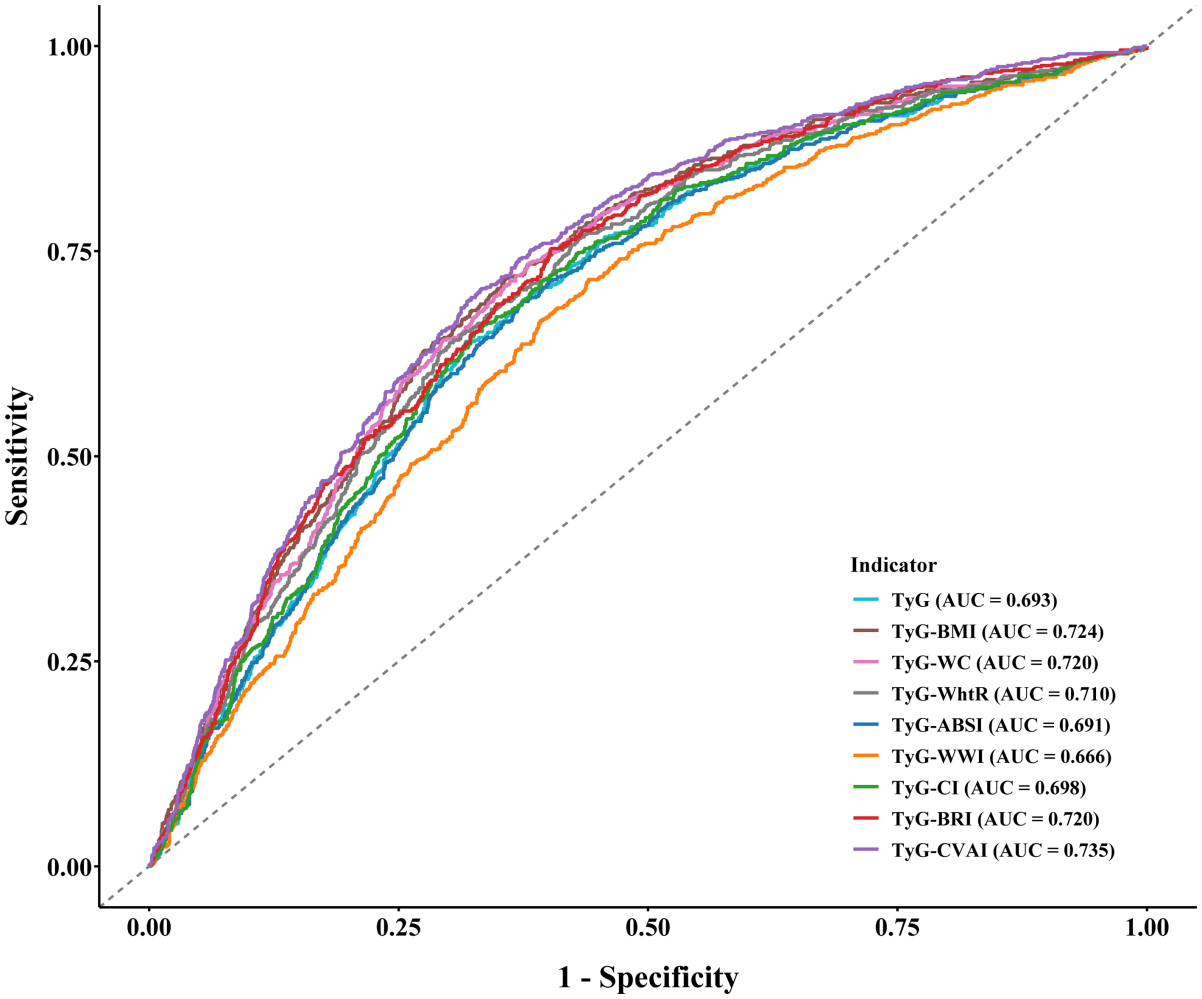
Comparison of AUC values of TyG-derived indices for predicting hyperuricemia.

A ROC analysis was performed to assess the discriminative performance of nine TyG-derived indicators in identifying hyperuricemia. Overall, these indices displayed moderate predictive capability, with AUC values spanning from approximately 0.666 to 0.735 (Figure 3). Among all indicators, TyG-CVAI showed the strongest performance, yielding an AUC of 0.735 (95% CI: 0.713–0.757). The predictive accuracy of TyG-BRI, TyG-BMI, and TyG-WC was slightly lower, with AUC values of 0.720 (95% CI: 0.697–0.742), 0.724 (95% CI: 0.702–0.746), and 0.720 (95% CI: 0.698–0.742), respectively. TyG-WhtR and TyG-CI demonstrated moderate discrimination, with AUCs of 0.710 (95% CI: 0.688–0.733) and 0.698 (95% CI: 0.676–0.721). In comparison, TyG-ABSI and TyG-WWI performed less favorably, with AUCs of 0.691 (95% CI: 0.668–0.714) and 0.666 (95% CI: 0.643–0.690). The TyG index alone achieved an AUC of 0.693 (95% CI: 0.670–0.716), indicating a modest discriminative ability.

Using TyG-CVAI as the reference, all remaining indicators demonstrated statistically significant reductions in discriminatory power (Supplementary Table S6). The largest performance gaps were observed for TyG-WWI and TyG-ABSI, which showed AUC differences of 0.069 (95% CI: 0.054–0.083) and 0.044 (95% CI: 0.033–0.054), respectively. More modest differences were noted for TyG-BRI (difference: 0.015, 95% CI: 0.010–0.021), TyG-BMI (difference: 0.011, 95% CI: 0.004–0.019), and TyG-WC (difference: 0.015, 95% CI: 0.008–0.022). Indicators reflecting glycemic–lipid metabolism alone, such as the TyG index (difference: 0.042, 95% CI: 0.031–0.053), also lagged behind TyG-CVAI. Despite variability across indices, all comparisons consistently confirmed that TyG-CVAI offered the most robust discriminative ability for hyperuricemia among the nine evaluated markers.

To further quantify the improvement in predictive performance of TyG-CVAI relative to the TyG index, NRI and IDI analyses were conducted (Supplementary Table S7). The NRI value was 0.421 (95% CI: 0.332–0.509, *p* < 0.001), suggesting that TyG-CVAI significantly improved the reclassification of individuals at risk of hyperuricemia by 42.1% compared with the TyG index. Moreover, the IDI value was 0.051 (95% CI: 0.039–0.062, *p* < 0.001), which indicated that TyG-CVAI enhanced the overall discriminatory capacity for hyperuricemia by an absolute 51% relative to the TyG index. These results collectively confirm that TyG-CVAI substantially strengthens the ability to stratify hyperuricemia risk beyond the traditional TyG index.

### 3.5 Sensitivity analyses

The robustness of the findings was further supported by multiple sensitivity analyses. First, imputing missing covariates produced estimates that were highly consistent with the primary findings (Supplementary Table S8). In addition, applying an alternative definition of hyperuricemia based on the Chinese clinical practice guideline did not materially alter the associations (Supplementary Table S9). The results also remained stable after removing participants with extreme TyG-derived index values (Supplementary Table S10). Collectively, these analyses demonstrated that the observed relationships were robust across different analytical assumptions.

## 4 Discussion

This study provides new evidence that a broad spectrum of TyG-derived adiposity indices—integrating metabolic dysfunction with body-fat distribution—are strongly associated with hyperuricemia in a working population. Across multiple analytic approaches, these indices consistently captured elevations in uric acid risk, revealing both monotonic and non-linear exposure–response patterns that highlight the complex metabolic disturbances underlying hyperuricemia. Notably, the TyG-CVAI emerged as the most informative indicator, outperforming all other TyG-based measures in discriminating hyperuricemia risk. These findings extend current knowledge on the metabolic relevance of TyG-related markers and underscore the value of combining biochemical and anthropometric information to enhance risk stratification. To our knowledge, this work represents the first comprehensive assessment comparing nine TyG-derived indices in relation to hyperuricemia, offering novel insights into their clinical and epidemiological utility within metabolic research.

Previous epidemiological studies have increasingly recognized the interplay between insulin resistance, adiposity, and hyperuricemia, yet most investigations have focused on either simple markers of insulin resistance, such as TyG or traditional anthropometric indicators such as BMI and waist circumference (21–24). Several population-based studies have reported positive associations between the TyG index and serum uric acid levels or hyperuricemia risk, and research on visceral adiposity indices such as BRI or CVAI has similarly suggested their relevance to urate dysregulation (23–25). However, existing studies typically examined these metrics in isolation, leaving important gaps regarding the comparative and combined utility of TyG-derived adiposity indices. Notably, no prior study has simultaneously evaluated a broad panel of TyG-related composite indicators within the same population, nor systematically compared their predictive performance for hyperuricemia. By integrating metabolic and anthropometric components, our study expands upon previous findings and provides the first comprehensive assessment of novel TyG-derived indices in a working population, thereby offering new insights into how distinct metabolic–adiposity phenotypes relate to uric acid metabolism.

The strong and consistent associations observed between multiple TyG-derived indices and hyperuricemia likely mirror shared pathophysiological pathways. As an established surrogate of insulin resistance, the TyG index reflects metabolic disturbances that promote oxidative stress, mitochondrial dysfunction, and inflammatory activation, ultimately suppressing urate excretory transporters while enhancing reabsorptive ones, thereby reducing uric acid clearance and contributing to hyperuricemia (9). Concurrently, visceral fat drives systemic inflammation, oxidative stress, and heightened purine turnover, all of which increase uric acid production (1, 26). By integrating metabolic and anthropometric components, the indices evaluated in this study may better capture both insulin-resistance–mediated and fat-distribution–related mechanisms than TyG alone. The particularly strong performance of TyG-CVAI may stem from its ability to reflect visceral adiposity patterns specific to Chinese populations, highlighting its value in assessing metabolic dysfunction and disturbances in uric acid regulation.

Our restricted cubic spline analyses revealed non-linear exposure–response relationships between the TyG-derived indices and hyperuricemia, indicating that risk does not increase in a purely linear fashion. For several indices, changes in the slope of risk were observed at higher values, suggesting that metabolic dysregulation may exert differential effects on uric acid levels across the exposure range. Such non-linear dynamics may reflect saturation of compensatory mechanisms or inflection points in renal urate handling. These patterns underscore the complexity of metabolic–uric acid interactions and suggest that early modest elevations in TyG-derived indices may carry lower incremental risk than more substantial increases, a finding with potential implications for risk stratification and intervention timing. Our subgroup analyses by age, sex, and shift-work status demonstrated that the associations between TyG-derived indices and hyperuricemia were remarkably stable across demographic and occupational strata, suggesting that these indices may be broadly applicable across different subpopulations. These findings support their use as screening tools in diverse working populations who may be at elevated metabolic risk.

Importantly, the observed associations may have particular relevance in the context of oilfield workers, whose occupational characteristics could exacerbate metabolic dysregulation and uric acid accumulation. Oilfield work is commonly characterized by irregular shift schedules, prolonged working hours, high physical demands, and frequent exposure to heat stress and dehydration. These factors have been associated with circadian disruption, impaired insulin sensitivity, and increased visceral adiposity, processes that are closely linked to elevated TyG-derived indices and altered urate metabolism. In such settings, TyG-CVAI may capture not only insulin resistance and central adiposity, but also the cumulative metabolic burden imposed by occupational stressors. Moreover, dehydration and heat exposure—common in oilfield environments—may reduce renal urate excretion and further amplify the impact of insulin resistance on serum uric acid levels. The strong performance of TyG-CVAI observed in this study, therefore, likely reflects a synergistic interaction between metabolic vulnerability and occupational exposures, underscoring its potential utility as a sensitive risk stratification tool in this specific workforce.

From a predictive standpoint, the marked superiority of TyG-CVAI over the other eight indices in ROC analysis suggests that it may serve as a practical and efficient marker for identifying individuals at high risk of hyperuricemia. In clinical settings, TyG-CVAI could be incorporated into routine metabolic risk assessment frameworks to identify individuals who may benefit from closer monitoring or early preventive strategies. Its ease of calculation and strong discriminative power make it a promising candidate for inclusion in metabolic-health screening programs, particularly in populations prone to visceral adiposity and insulin resistance. In addition to its etiological implications, the present findings also carry important relevance for occupational health practice. In occupational settings characterized by elevated metabolic risk, TyG-derived indices—particularly TyG-CVAI—may serve as practical tools for early risk identification and targeted prevention. Because TyG-CVAI is derived entirely from routinely collected anthropometric and biochemical parameters, it could be readily incorporated into regular occupational health examinations without additional cost or burden. Individuals identified in the higher distribution ranges of TyG-CVAI may benefit from closer metabolic monitoring and targeted preventive strategies, such as lifestyle counseling focused on weight management, dietary modification, and physical activity promotion. From a population health perspective, integrating TyG-CVAI into existing occupational screening programs may facilitate earlier identification of workers at elevated risk for hyperuricemia and related metabolic disorders, thereby supporting timely and individualized preventive interventions in this high-risk occupational group.

Our study features a comprehensive evaluation of TyG-related indicators and rigorous statistical modeling. However, our findings must be interpreted in light of certain limitations. First, the cross-sectional design precludes causal inference; it remains unclear whether elevated TyG-derived indices contribute directly to uric acid accumulation or simply correlate with it. Prospective cohort studies are warranted to determine whether longitudinal changes in TyG-derived indices precede the development of hyperuricemia. Second, participants were recruited from a single oilfield enterprise in northwest China, with a predominantly Han population, which may limit the generalizability of our findings to other regions, ethnic groups, or occupational settings. Further validation in multi-center cohorts encompassing diverse ethnic backgrounds and occupational environments is required to determine whether the observed associations are consistent beyond oilfield workers. Third, key factors influencing uric acid metabolism, such as detailed dietary intake of purine- and fructose-rich foods and the use of diuretics, were not measured, and residual confounding cannot be fully excluded. Fourth, although the study population consisted of oilfield enterprise employees, detailed task-specific occupational classifications were not available, and heterogeneity in work intensity and exposure profiles may have introduced residual confounding. Finally, while TyG-derived indices offer convenience and low cost, future studies should compare them with gold-standard measures such as imaging-based visceral adiposity assessments or hyperinsulinemic-euglycemic clamp data.

## 5 Conclusion

In conclusion, our study provides novel, clinically relevant insights into the metabolic underpinnings of hyperuricemia. By demonstrating robust associations between multiple TyG-derived adiposity indices and hyperuricemia, as well as identifying TyG-CVAI as the most predictive marker, we highlight the potential of these indices for early detection of uric acid-related risk in working populations. Future prospective and interventional research is needed to validate their predictive utility and to explore whether modification of these indices through lifestyle or pharmacological measures can reduce hyperuricemia and its downstream consequences.

## Data Availability

The raw data supporting the conclusions of this article will be made available by the authors, without undue reservation.
