# Supplementary material for: Association of novel triglyceride-glucose index-derived indices with hyperuricemia among oilfield workers
Source: Front Nutr. 2026 Feb 6;13:1766524. doi: 10.3389/fnut.2026.1766524 (PMC12920181; doi:10.3389/fnut.2026.1766524)
Supplement: Supplementary file 1 [file Data_Sheet_1.PDF]

Supplementary material

Table S1. Calculation formulas of the nine TyG-derived indices

Table S2. Category and definition of covariates.

Table S3. Stratified analyses by age for the associations between TyG-derived indices and the risk of hyperuricemia among oilfield workers.

Table S4. Stratified analyses by sex for the associations between TyG-derived indices and the risk of hyperuricemia among oilfield workers.

Table S5. Stratified analyses by shift work for the associations between TyG-derived indices and the risk of hyperuricemia among oilfield workers.

Table S6. Comparison of AUC values of TyG-derived indices for predicting hyperuricemia.

Table S7. Net reclassification improvement and integrated discrimination improvement for comparing the predictive performance of TyG and TyG-CVAI in risk of hyperuricemia among oilfield workers.

Table S8. Associations between TyG-derived indices and the risk of hyperuricemia among oilfield workers, after multiple imputation for missing covariates.

Table S9. Associations between TyG-derived indices and the risk of hyperuricemia among oilfield workers, using the Chinese clinical guideline definition of hyperuricemia.

Table S10. Associations between TyG-derived indices and the risk of hyperuricemia among oilfield workers, after exclusion of participants with extreme index values.

Table S1. Calculation formulas of the nine TyG-derived indices

Indicator	Formula
TyG	ln (TG × FPG/2)
TyG-BMI	TyG × BMI
TyG-WC	TyG × WC
TyG-WhtR	TyG × (WC / height)
TyG-ABSI	TyG × ABSI, where ABSI = WC / (BMI^(2/3) × height^(1/2))
TyG-WWI	TyG × WWI, where WWI = WC / sqrt(weight)
TyG-CI	TyG × CI, where CI = WC / [0.109 × sqrt(weight / height)]
TyG-BRI	TyG × BRI, where BRI = 364.2 − 365.5 × sqrt[1 − (WC / (2π))^2 / (0.5 × height)^2]
TyG-CVAI	TyG × CVAI, where CVAI (male) = −267.93 + 0.68 × age + 0.03 × BMI + 4.00 × WC + 22.00 × log10 TG − 16.32 × HDL-C; CVAI (female) = −187.32 + 1.71 × age + 4.23 × BMI + 1.12 × WC + 39.76 × log10 TG − 11.66 × HDL-C

Table S2. Category and definition of covariates.

Variable	Category and definition
Shift work	Shift work is defined as the regular rotation of individuals to work outside the hours of 8:00 AM to 5:00 PM for a minimum duration of one year.
Chemical substance exposure	Chemical substance exposure is defined as the self-reported exposure of individuals to hazardous chemical substances in the workplace or environment, including benzene, toluene, xylene, hydrogen sulfide, carbon monoxide, nitrogen oxides, carbon tetrachloride, n-hexane, n-pentane, gasoline, etc.
Noise exposure	Noise exposure is defined as the self-reported exposure of individuals to noise levels in the work environment that exceed legal noise standards.
Dust exposure	Dust exposure is defined as the self-reported exposure of individuals to inhalable or respirable dust particles during the work process, which may originate from production processes, material handling, or the surrounding environment.
Cigarette smoking	Cigarette smoking is defined as individuals who smoke at least one cigarette daily for six months or longer.
Alcohol drinking	Alcohol drinking is defined as those who consume alcohol at least once a week and maintain this frequency for six months or longer.
Physical activity	Physical activity is assessed based on questionnaire items asking participants whether they usually engage in moderate-intensity activities—such as slow jogging or moderate-paced cycling—lasting at least 10 minutes per session.
Tea drinking	Tea drinking is defined as consuming tea at least three times per week for a duration of six months or longer.
Estimated glomerular filtration rate	The estimated glomerular filtration rate is calculated to evaluate renal function, which is derived using the Chronic Kidney Disease Epidemiology Collaboration equation.
Hypertension	Hypertension is defined as systolic blood pressure ≥140 mmHg, diastolic blood pressure ≥90 mmHg, self-reported physician diagnosis of hypertension, or current use of antihypertensive medication.
Hyperlipidemia	Hyperlipidemia is defined as total cholesterol ≥6.2 mmol/L, triglycerides ≥2.3 mmol/L, low-density lipoprotein cholesterol ≥4.1 mmol/L, high-density lipoprotein cholesterol <1.0 mmol/L, self-reported physician diagnosis of hyperlipidemia, or current use of lipid-lowering medication.
Cardiovascular disease	Cardiovascular disease is defined as a self-reported history of coronary heart disease, atherosclerosis, or stroke.

Table S3. Stratified analyses by age for the associations between TyG-derived indices and the risk of hyperuricemia among oilfield workers.

Indicators	OR (95 % CI)	*P* value
**Age, <40 years**		
TyG-ABSI	1.58 (1.28, 1.96)	<0.001
TyG-WWI	1.34 (1.10, 1.64)	0.004
TyG-CI	1.68 (1.36, 2.09)	<0.001
TyG-BRI	2.03 (1.63, 2.56)	<0.001
TyG-CVAI	2.18 (1.71, 2.80)	<0.001
**Age, ≥40 years**		
TyG-ABSI	1.58 (1.35, 1.86)	<0.001
TyG-WWI	1.48 (1.27, 1.74)	<0.001
TyG-CI	1.61 (1.37, 1.90)	<0.001
TyG-BRI	1.64 (1.41, 1.93)	<0.001
TyG-CVAI	1.64 (1.40, 1.93)	<0.001

The models were adjusted for sex, ethnicity, education level, marital status, annual income, shift work, chemical substance exposure, noise exposure, dust exposure, cigarette smoking, alcohol drinking, tea drinking, physical activity, estimated glomerular filtration rate, hypertension, hyperlipidemia, and cardiovascular disease. OR, odds ratio; CI, confidence interval.

Table S4. Stratified analyses by sex for the associations between TyG-derived indices and the risk of hyperuricemia among oilfield workers.

Indicators	OR (95 % CI)	*P* value
**Male**		
TyG-ABSI	1.58 (1.38, 1.82)	<0.001
TyG-WWI	1.42 (1.24, 1.63)	<0.001
TyG-CI	1.63 (1.42, 1.88)	<0.001
TyG-BRI	1.81 (1.56, 2.10)	<0.001
TyG-CVAI	1.81 (1.57, 2.10)	<0.001
**Female**		
TyG-ABSI	1.54 (1.16, 2.05)	0.003
TyG-WWI	1.42 (1.08, 1.87)	0.014
TyG-CI	1.59 (1.20, 2.12)	0.001
TyG-BRI	1.72 (1.32, 2.27)	<0.001
TyG-CVAI	1.89 (1.43, 2.53)	<0.001

The models were adjusted for age, ethnicity, education level, marital status, annual income, shift work, chemical substance exposure, noise exposure, dust exposure, cigarette smoking, alcohol drinking, tea drinking, physical activity, estimated glomerular filtration rate, hypertension, hyperlipidemia, and cardiovascular disease. OR, odds ratio; CI, confidence interval.

Table S5. Stratified analyses by shift work for the associations between TyG-derived indices and the risk of hyperuricemia among oilfield workers.

Indicators	OR (95 % CI)	*P* value
**Non-shift work**		
TyG-ABSI	1.89 (1.47, 2.45)	<0.001
TyG-WWI	1.61 (1.26, 2.06)	<0.001
TyG-CI	1.98 (1.54, 2.58)	<0.001
TyG-BRI	2.14 (1.68, 2.78)	<0.001
TyG-CVAI	2.29 (1.77, 3.01)	<0.001
**Shift work**		
TyG-ABSI	1.57 (1.35, 1.83)	<0.001
TyG-WWI	1.43 (1.24, 1.67)	<0.001
TyG-CI	1.61 (1.39, 1.88)	<0.001
TyG-BRI	1.74 (1.49, 2.03)	<0.001
TyG-CVAI	1.77 (1.51, 2.09)	<0.001

The models were adjusted for age, sex, ethnicity, education level, marital status, annual income, chemical substance exposure, noise exposure, dust exposure, cigarette smoking, alcohol drinking, tea drinking, physical activity, estimated glomerular filtration rate, hypertension, hyperlipidemia, and cardiovascular disease. OR, odds ratio; CI, confidence interval.

Table S6. Comparison of AUC values of TyG-derived indices for predicting hyperuricemia.

Indicators	AUC (95 % CI)	Difference (95 % CI)#	*P* value#
TyG-CVAI	0.735 (0.713, 0.757)	—	—
TyG-ABSI	0.691 (0.668, 0.714)	0.044 (0.033, 0.054)	<0.001
TyG-WWI	0.666 (0.643, 0.690)	0.069 (0.054, 0.083)	<0.001
TyG-CI	0.698 (0.676, 0.721)	0.037 (0.027, 0.046)	<0.001
TyG-BRI	0.720 (0.697, 0.742)	0.015 (0.010, 0.021)	<0.001
TyG-BMI	0.724 (0.702, 0.746)	0.011 (0.004, 0.019)	0.003
TyG-WC	0.720 (0.698, 0.742)	0.015 (0.008, 0.022)	<0.001
TyG-WhtR	0.710 (0.688, 0.733)	0.025 (0.017, 0.032)	<0.001
TyG	0.693 (0.670, 0.716)	0.042 (0.031, 0.053)	<0.001

#, compared with TyG-CVAI.

Table S7. Net reclassification improvement and integrated discrimination improvement for comparing the predictive performance of TyG and TyG-CVAI in risk of hyperuricemia among oilfield workers.

Metric	Value	95% Confidence Interval	*P* value
Net reclassification improvement (NRI)	0.421	0.332 to 0.509	<0.001
Integrated Discrimination Improvement (IDI)	0.051	0.039 to 0.062	<0.001

Table S8. Associations between TyG-derived indices and the risk of hyperuricemia among oilfield workers, after multiple imputation for missing covariates.

Variable	Model 1		Model 2		Model 3	
	OR (95 % CI)	*P* value	OR (95 % CI)	*P* value	OR (95 % CI)	*P* value
**TyG-ABSI**						
Per SD increase	1.86 (1.71, 2.01)	<0.001	1.74 (1.59, 1.90)	<0.001	1.66 (1.48, 1.85)	<0.001
Quantile 1	1.00 (Reference)		1.00 (Reference)		1.00 (Reference)	
Quantile 2	1.77 (1.34, 2.35)	<0.001	1.64 (1.22, 2.21)	0.001	1.62 (1.20, 2.19)	0.002
Quantile 3	3.97 (3.07, 5.17)	<0.001	3.74 (2.83, 4.99)	<0.001	3.54 (2.63, 4.81)	<0.001
Quantile 4	6.19 (4.81, 8.04)	<0.001	5.27 (3.99, 7.02)	<0.001	4.82 (3.44, 6.79)	<0.001
P for trend		<0.001		<0.001		<0.001
**TyG-WWI**						
Per SD increase	1.71 (1.58, 1.86)	<0.001	1.61 (1.47, 1.76)	<0.001	1.48 (1.33, 1.65)	<0.001
Quantile 1	1.00 (Reference)		1.00 (Reference)		1.00 (Reference)	
Quantile 2	1.81 (1.38, 2.37)	<0.001	1.66 (1.25, 2.20)	<0.001	1.57 (1.18, 2.11)	0.002
Quantile 3	3.53 (2.75, 4.56)	<0.001	3.26 (2.48, 4.30)	<0.001	2.96 (2.22, 3.96)	<0.001
Quantile 4	4.98 (3.90, 6.42)	<0.001	4.23 (3.23, 5.58)	<0.001	3.40 (2.46, 4.71)	<0.001
P for trend		<0.001		<0.001		<0.001
**TyG-CI**						
Per SD increase	1.91 (1.76, 2.07)	<0.001	1.7 (1.63, 1.95)8	<0.001	1.71 (1.53, 1.92)	<0.001
Quantile 1	1.00 (Reference)		1.00 (Reference)		1.00 (Reference)	
Quantile 2	1.75 (1.32, 2.32)	<0.001	1.64 (1.22, 2.22)	0.001	1.63 (1.21, 2.22)	0.002
Quantile 3	4.07 (3.14, 5.31)	<0.001	3.77 (2.85, 5.04)	<0.001	3.67 (2.72, 4.98)	<0.001
Quantile 4	6.57 (5.09, 8.54)	<0.001	5.66 (4.27, 7.55)	<0.001	5.37 (3.82, 7.60)	<0.001
P for trend		<0.001		<0.001		<0.001
**TyG-BRI**						
Per SD increase	2.02 (1.86, 2.20)	<0.001	1.90 (1.74, 2.09)	<0.001	1.85 (1.66, 2.08)	<0.001
Quantile 1	1.00 (Reference)		1.00 (Reference)		1.00 (Reference)	
Quantile 2	1.85 (1.38, 2.49)	<0.001	1.88 (1.38, 2.58)	<0.001	1.79 (1.30, 2.47)	<0.001
Quantile 3	6.08 (4.73, 7.91)	<0.001	5.77 (4.36, 7.71)	<0.001	5.23 (3.84, 7.20)	<0.001
Quantile 4	6.13 (4.58, 8.28)	<0.001	6.33 (4.57, 8.82)	<0.001	5.42 (3.82, 7.75)	<0.001
P for trend		<0.001		<0.001		<0.001
**TyG-CVAI**						
Per SD increase	2.13 (1.96, 2.32)	<0.001	1.98 (1.80, 2.19)	<0.001	1.94 (1.72, 2.18)	<0.001
Quantile 1	1.00 (Reference)		1.00 (Reference)		1.00 (Reference)	
Quantile 2	2.00 (1.48, 2.71)	<0.001	2.10 (1.52, 2.91)	<0.001	1.97 (1.42, 2.75)	<0.001
Quantile 3	4.39 (3.34, 5.85)	<0.001	4.56 (3.32, 6.32)	<0.001	4.32 (3.08, 6.11)	<0.001
Quantile 4	10.00 (7.65, 13.23)	<0.001	9.80 (7.13, 13.61)	<0.001	9.52 (6.57, 13.94)	<0.001
P for trend		<0.001		<0.001		<0.001

Model 1, no covariate was adjusted. Model 2, adjusted for age, sex, ethnicity, education level, marital status, and annual income. Model 3, further adjusted for body mass index, shift work, chemical substance exposure, noise exposure, dust exposure, cigarette smoking, alcohol drinking, tea drinking, physical activity, estimated glomerular filtration rate, hypertension, hyperlipidemia, and cardiovascular disease. OR, odds ratio; CI, confidence interval.

Table S9. Associations between TyG-derived indices and the risk of hyperuricemia among oilfield workers, using the Chinese clinical guideline definition of hyperuricemia.

Variable	Model 1		Model 2		Model 3	
	**OR (95 % CI)**	***P* value**	**OR (95 % CI)**	***P* value**	**OR (95 % CI)**	***P* value**
**TyG-ABSI**						
Per SD increase	1.94 (1.76, 2.14)	<0.001	1.70 (1.53, 1.90)	<0.001	1.65 (1.44, 1.88)	<0.001
Quantile 1	1.00 (Reference)		1.00 (Reference)		1.00 (Reference)	
Quantile 2	2.28 (1.59, 3.33)	<0.001	1.77 (1.20, 2.65)	0.005	1.79 (1.21, 2.69)	0.004
Quantile 3	5.07 (3.62, 7.25)	<0.001	3.94 (2.72, 5.79)	<0.001	3.85 (2.62, 5.74)	<0.001
Quantile 4	8.42 (6.05, 11.95)	<0.001	5.59 (3.88, 8.19)	<0.001	5.42 (3.54, 8.41)	<0.001
P for trend		<0.001		<0.001		<0.001
**TyG-WWI**						
Per SD increase	1.78 (1.62, 1.96)	<0.001	1.58 (1.42, 1.76)	<0.001	1.48 (1.30, 1.69)	<0.001
Quantile 1	1.00 (Reference)		1.00 (Reference)		1.00 (Reference)	
Quantile 2	2.12 (1.50, 3.01)	<0.001	1.70 (1.18, 2.48)	0.005	1.62 (1.12, 2.37)	0.011
Quantile 3	4.24 (3.08, 5.92)	<0.001	3.26 (2.30, 4.67)	<0.001	3.02 (2.10, 4.39)	<0.001
Quantile 4	6.03 (4.40, 8.37)	<0.001	4.19 (2.98, 5.98)	<0.001	3.51 (2.35, 5.28)	<0.001
P for trend		<0.001		<0.001		<0.001
**TyG-CI**						
Per SD increase	1.99 (1.81, 2.20)	<0.001	1.74 (1.56, 1.94)	<0.001	1.70 (1.49, 1.95)	<0.001
Quantile 1	1.00 (Reference)		1.00 (Reference)		1.00 (Reference)	
Quantile 2	2.37 (1.63, 3.49)	<0.001	1.87 (1.26, 2.83)	0.002	1.90 (1.27, 2.87)	0.002
Quantile 3	5.50 (3.89, 7.92)	<0.001	4.20 (2.88, 6.25)	<0.001	4.20 (2.84, 6.33)	<0.001
Quantile 4	9.35 (6.67, 13.40)	<0.001	6.29 (4.33, 9.30)	<0.001	6.30 (4.08, 9.88)	<0.001
P for trend		<0.001		<0.001		<0.001
**TyG-BRI**						
Per SD increase	2.06 (1.87, 2.28)	<0.001	1.86 (1.66, 2.08)	<0.001	1.83 (1.60, 2.09)	<0.001
Quantile 1	1.00 (Reference)		1.00 (Reference)		1.00 (Reference)	
Quantile 2	2.40 (1.62, 3.62)	<0.001	2.15 (1.41, 3.33)	<0.001	2.11 (1.38, 3.26)	<0.001
Quantile 3	5.89 (4.11, 8.66)	<0.001	5.09 (3.42, 7.74)	<0.001	5.29 (3.50, 8.15)	<0.001
Quantile 4	12.30 (8.65, 17.94)	<0.001	9.48 (6.41, 14.34)	<0.001	10.02 (6.44, 15.87)	<0.001
P for trend		<0.001		<0.001		<0.001
**TyG-CVAI**						
Per SD increase	2.28 (2.06, 2.53)	<0.001	1.87 (1.68, 2.10)	<0.001	1.85 (1.62, 2.12)	<0.001
Quantile 1	1.00 (Reference)		1.00 (Reference)		1.00 (Reference)	
Quantile 2	3.00 (1.96, 4.69)	<0.001	2.21 (1.40, 3.58)	<0.001	2.02 (1.28, 3.26)	0.003
Quantile 3	7.68 (5.20, 11.74)	<0.001	5.12 (3.30, 8.15)	<0.001	4.67 (2.98, 7.49)	<0.001
Quantile 4	16.61 (11.33, 25.21)	<0.001	9.65 (6.26, 15.32)	<0.001	9.01 (5.59, 14.87)	<0.001
P for trend		<0.001		<0.001		<0.001

Model 1, no covariate was adjusted. Model 2, adjusted for age, sex, ethnicity, education level, marital status, and annual income. Model 3, further adjusted for body mass index, shift work, chemical substance exposure, noise exposure, dust exposure, cigarette smoking, alcohol drinking, tea drinking, physical activity, estimated glomerular filtration rate, hypertension, hyperlipidemia, and cardiovascular disease. OR, odds ratio; CI, confidence interval.

Table S10. Associations between TyG-derived indices and the risk of hyperuricemia among oilfield workers, after exclusion of participants with extreme index values.

Variable	Model 1		Model 2		Model 3	
	OR (95 % CI)	*P* value	OR (95 % CI)	*P* value	OR (95 % CI)	*P* value
**TyG-ABSI**						
Per SD increase	2.04 (1.84, 2.26)	<0.001	1.92(1.71, 2.15)	<0.001	1.80 (1.56, 2.08)	<0.001
Quantile 1	1.00 (Reference)		1.00 (Reference)		1.00 (Reference)	
Quantile 2	1.85 (1.33, 2.59)	<0.001	1.68 (1.19, 2.39)	0.004	1.66 (1.16, 2.38)	0.005
Quantile 3	3.83 (2.82, 5.25)	<0.001	3.64 (2.61, 5.12)	<0.001	3.38 (2.38, 4.84)	<0.001
Quantile 4	6.46 (4.80, 8.81)	<0.001	5.51 (3.96, 7.75)	<0.001	4.76 (3.21, 7.14)	<0.001
P for trend		<0.001		<0.001		<0.001
**TyG-WWI**						
Per SD increase	1.81 (1.64, 2.00)	<0.001	1.70 (1.53, 1.90)	<0.001	1.53 (1.33, 1.75)	<0.001
Quantile 1	1.00 (Reference)		1.00 (Reference)		1.00 (Reference)	
Quantile 2	1.81(1.32, 2.50)	<0.001	1.68 (1.20, 2.36)	0.003	1.57 (1.11, 2.22)	0.011
Quantile 3	3.53 (2.63, 4.78)	<0.001	3.23 (2.34, 4.48)	<0.001	2.89 (2.06, 4.08)	<0.001
Quantile 4	5.04 (3.77, 6.80)	<0.001	4.38 (3.18, 6.08)	<0.001	3.35 (2.29, 4.93)	<0.001
P for trend		<0.001		<0.001		<0.001
**TyG-CI**						
Per SD increase	2.10 (1.90, 2.34)	<0.001	1.98 (1.76, 2.22)	<0.001	1.89 (1.63, 2.18)	<0.001
Quantile 1	1.00 (Reference)		1.00 (Reference)		1.00 (Reference)	
Quantile 2	1.84 (1.32, 2.60)	<0.001	1.70 (1.19, 2.43)	0.004	1.67 (1.16, 2.40)	0.006
Quantile 3	4.10 (3.01, 5.65)	<0.001	3.86 (2.76, 5.47)	<0.001	3.65 (2.56, 5.26)	<0.001
Quantile 4	7.09 (5.24, 9.71)	<0.001	6.10 (4.36, 8.63)	<0.001	5.47 (3.67, 8.24)	<0.001
P for trend		<0.001		<0.001		<0.001
**TyG-BRI**						
Per SD increase	2.33 (2.09, 2.60)	<0.001	2.23 (1.98, 2.52)	<0.001	2.22 (1.92, 2.58)	<0.001
Quantile 1	1.00 (Reference)		1.00 (Reference)		1.00 (Reference)	
Quantile 2	2.03 (1.43, 2.91)	<0.001	2.12 (1.46, 3.11)	<0.001	2.07 (1.41, 3.05)	<0.001
Quantile 3	4.39 (3.18, 6.16)	<0.001	4.71 (3.29, 6.82)	<0.001	4.66 (3.20, 6.88)	<0.001
Quantile 4	9.31 (6.80, 12.96)	<0.001	9.17 (6.44, 13.25)	<0.001	8.99 (5.98, 13.68)	<0.001
P for trend		<0.001		<0.001		<0.001
**TyG-CVAI**						
Per SD increase	2.39 (2.15, 2.66)	<0.001	2.26 (2.00, 2.56)	<0.001	2.26 (1.95, 2.64)	<0.001
Quantile 1	1.00 (Reference)		1.00 (Reference)		1.00 (Reference)	
Quantile 2	1.99 (1.39, 2.87)	<0.001	2.11 (1.43, 3.12)	<0.001	1.93 (1.31, 2.88)	0.001
Quantile 3	4.83 (3.49, 6.81)	<0.001	5.07 (3.49, 7.47)	<0.001	4.49 (3.02, 6.75)	<0.001
Quantile 4	9.58 (6.97, 13.41)	<0.001	9.41 (6.46, 13.94)	<0.001	8.36 (5.42, 13.07)	<0.001
P for trend		<0.001		<0.001		<0.001

Model 1, no covariate was adjusted. Model 2, adjusted for age, sex, ethnicity, education level, marital status, and annual income. Model 3, further adjusted for body mass index, shift work, chemical substance exposure, noise exposure, dust exposure, cigarette smoking, alcohol drinking, tea drinking, physical activity, estimated glomerular filtration rate, hypertension, hyperlipidemia, and cardiovascular disease. OR, odds ratio; CI, confidence interval.
